# Raman spectroscopy determination of the mineral characteristics of microcalcifications in breast cancer: a way towards an improved screening approach

**DOI:** 10.18632/oncotarget.27912

**Published:** 2021-05-11

**Authors:** Fabio Corsi, Carlo Morasso

**Keywords:** Raman spectroscopy, microcalcifications, breast cancer

Breast cancer (BC) is the most frequently diagnosed malignancy in women and currently, it is expected that 1 woman every 10 will receive a diagnosis of BC during her lifetime in countries with high sociodemographic index [1]. Thanks to the wide adoption of screening mammography, now the vast majority (90–95%) of BC are diagnosed at an early stage and the five-year survival rate of BC exceeds 80% [2].

During mammography screening, the detection of microcalcification (MCs) is common; and MCs are an important early hallmark of BC. On the other hand, only a small proportion of MCs are associated with the presence of BC, supporting the fact that screening mammography is highly sensitive but poorly specific [3]. The improvement of BC screening programs, with the final aim to reduce the number of unnecessary biopsies and other invasive procedures to role out malignancy, is, therefore, an unmet clinical need. A possible solution could be the improvement of our knowledge about the mineral characteristic of BC-associated MCs.

Raman Spectroscopy (RS) is a photonic approach that allows a label-free characterization of mineral samples in real-time and virtually without the need to use any sample preparation protocol [4]. RS is based on the analysis of the light inelastically scattered by a sample under the illumination of a monochromatic laser light source. Because of the molecular vibrations, part of the energy of the incoming light is held by the samples and thus a small portion of the light is scattered with a lower amount of energy (thus with a different wavelength) whose frequency depends on the nature of the sample itself. RS is particularly well suited for the study of the crystal properties of inorganic materials, as proved by the fact that is used as a technique for quality control in the pharmaceutical and semiconductor industry [5].

A first report from Haka et al. in 2002 [6] demonstrated how RS is univocally able to distinguish between calcium oxalate associated with benign lesions) and calcium phosphate (both in malign and benign lesions) MCs. Furthermore, RS was able to distinguish between malign and benign MCs with a 93% specificity. This impressive result sparked a notable interest and several groups started working on the development of Raman approaches able to provide a non-invasive, or minimally invasive, tool for the analysis of MCs *in vivo* or *ex vivo* on biopsies immediately after the excision [7–9].

This technological work, however, is based on thin clinical data. The report from Haka and co-workers included in the analysis just 90 MCs from 11 patients, and larger studies on the effectiveness of RS in distinguishing between MCs associated with benign and malignant lesions are needed.

Recently, we started a research study of 473 MCs biopsies performed after screening mammography in a larger cohort of 56 patients [10]. Our results confirmed, on a larger scale, the original report from Haka. Remarkably, we also studied the composition of MCs from BCs but located outside the malignant lesion and we found that the characteristics of these MCs are similar to those located inside the lesion. This result strengthens the idea that RS on MCs could be a way to improve the accuracy of mammography screening as suggests that Raman analysis of MCs would not strictly require focusing only on MCs within the tumor to provide clinically meaningful information.

The work done until now, however, must still be improved. To prove the clinical value of RS, we need to study an even larger cohort of patients. Besides, up to now, no attempt has been performed yet to classify patients based on RS data.

In the next future, this work will be merged with the development of new classification algorithms and with new tools for the acquisition of the spectra to deliver an advanced mammogram, able to identify in real-time BC lesions with a much-improved accuracy. This will allow to finally reduce the economical and psychological burden of false-positive mammography with a great benefit for both the patients and the healthcare system.
